# Supplementary material for: A fault-intrusion-tolerant system and deadline-aware algorithm for scheduling scientific workflow in the cloud
Source: PeerJ Comput Sci. 2021 Nov 2;7:e747. doi: 10.7717/peerj-cs.747 (PMC8576554; doi:10.7717/peerj-cs.747)
Supplement: Supplemental Information 6 [file peerj-cs-07-747-s006.zip › Myproject1.1/docs/org/workflowsim/failure/package-summary.html]

org.workflowsim.failure


JavaScript is disabled on your browser.


- Overview
- Package
- Class
- Use
- Tree
- Deprecated
- Index
- Help

- Prev Package
- Next Package

- Frames
- No Frames

- All Classes

# Package org.workflowsim.failure

- Class Summary

  | Class | Description |
  | --- | --- |
  |  |  |
  | --- | --- |
  | FailureGenerator | FailureGenerator creates a failure when a job returns |
  | FailureMonitor | FailureMonitor collects failure information |
  | FailureRecord | Failure Record is a record with all information of a failure |

- Overview
- Package
- Class
- Use
- Tree
- Deprecated
- Index
- Help

- Prev Package
- Next Package

- Frames
- No Frames

- All Classes
